# Exploring the Effect of Cetylpyridinium Chloride Addition on the Antibacterial Activity and Surface Hardness of Resin-Based Dental Composites

**DOI:** 10.3390/polym16050588

**Published:** 2024-02-21

**Authors:** Sara Khan, Faiza Amin, Rafat Amin, Naresh Kumar

**Affiliations:** 1 Department of Science of Dental Materials, Dr. Ishrat Ul Ebad Khan Institute of Oral Health Sciences, Dow University of Health Sciences, Karachi 74200, Pakistan; sara.khan17@dikiohs.duhs.edu.pk (S.K.); kumar.naresh@duhs.edu.pk (N.K.); 2 Department of Science of Dental Materials, Dow Dental College, Dow University of Health Sciences, Karachi 74200, Pakistan; 3 Dow College of Biotechnology, Dow University of Health Sciences, Karachi 74200, Pakistan; rafat.amin@duhs.edu.pk

**Keywords:** resin-based dental composite, cetylpyridinium chloride, antibacterial activity, surface hardness, agar diffusion test, direct contact test

## Abstract

The aim of this study was to evaluate the effect of cetylpyridinium chloride (CPC) addition on the antibacterial and surface hardness characteristics of two commercial resin-based dental composites (RBDCs). A total of two hundred and seventy (*n* = 270) specimens from Filtek Z250 Universal and Filtek Z350 XT flowable RBDCs were fabricated with the addition of CPC at 2 %wt and 4 %wt concentrations to assess their antibacterial activity using the agar diffusion test and direct contact inhibition test, and their surface hardness using the Vickers microhardness test after 1 day, 30 days, and 90 days of aging. A surface morphology analysis of the specimens was performed using a scanning electron microscope (SEM). The RBDCs that contained 2 %wt and 4 %wt CPC demonstrated significant antibacterial activity against *Streptococcus mutans* up to 90 days, with the highest activity observed for the 4 %wt concentration. Nevertheless, there was a reduction in antibacterial effectiveness over time. Moreover, compared to the control (0 %wt) and 2 %wt CPC groups, the universal RBDCs containing 4 %wt CPC exhibited a notable decrease in surface hardness, while all groups showed a decline in hardness over time. In conclusion, the satisfactory combination of the antibacterial effect and surface hardness property of RBDCs was revealed with the addition of a 2 %wt CPC concentration.

## 1. Introduction

Globally, dental caries in permanent teeth ranks as the 10th most prevalent human disease [1]. Dental caries is a complex condition with multiple causes [2], and secondary caries leading to restoration replacement is a significant issue, accounting for 50–60% of restorations in both permanent and primary dentition [3]. According to a randomized control trial, in the U.S, about USD 5 billion is spent annually on restoration replacement, with 87.6% of resin-based dental composites (RBDC) and 66.7% of amalgam restorations facing clinical failure due to secondary caries [4]. RBDCs offer advantages like excellent esthetics, quick placement, a high bond strength, and tissue preservation [5]. However, their concerns include polymerization shrinkage and micro-leakage, which may lead to bacterial accumulation, causing secondary caries [6,7]. Moreover, RBDCs tend to accumulate plaque due to surface roughness and residual monomer release [8,9]. In order to overcome this problem, modern-day research is playing its role in creating bacterial inhibiting restorative filling materials that prevent recurrent decay and, eventually, restoration failure [10].

Bacteria represent the predominant microorganisms in the human oral cavity. Gram-positive bacteria, including *Streptococcus mutans*, *Lactobacillus*, and *Actinomycetes* are frequently associated with dental caries due to their acidogenic and aciduric nature. Among them, *Streptococcus mutans* holds particular importance, while *Lactobacillus*, although not an initiator, plays a crucial role in caries progression [11]. 

Oral biofilm formation progresses through stages, including pathogenic microorganism entry, microflora congregation, biofilm-forming bacteria increase leading to dysbiosis, drug-resistant gene exchange, and biofilm maturation. The dynamic biofilm environment contributes to an increased susceptibility to secondary caries development in proximity to restorations [12].

**Figure 1:**
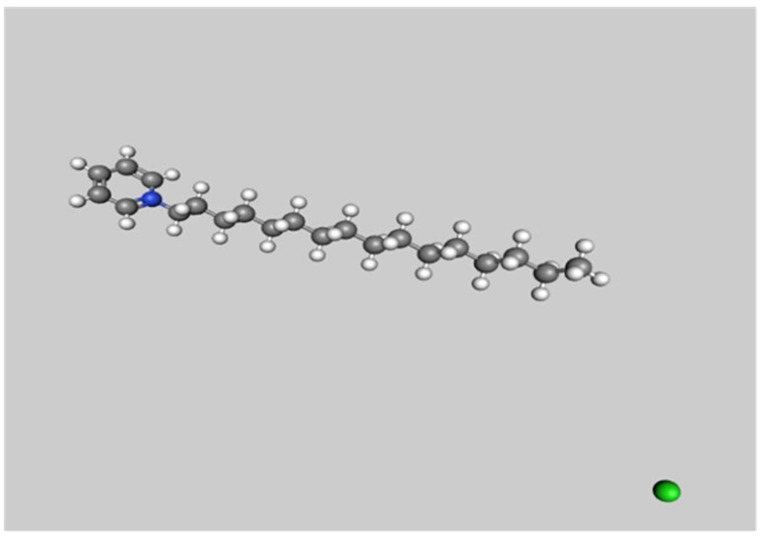
Illustration of molecular structure of cetylpyridinium chloride. Atomic hues include grey for carbon, white for hydrogen, blue for nitrogen, and green for chlorine.

The chemical composition of CPC consists of the cetylpyridinium ion and chloride salt with the molecular formula C_21_H_38_ClN and a molecular weight of 340 g/mol; it is characterized as a white, non-hygroscopic, dry powder (Figure 1). This compound exhibits a high solubility in water and various organic solvents, including alcohol and chloroform. In aqueous solutions, it maintains a pH range of 6.0–7.0 [13]. CPC’s amphiphilic nature enables it to inhibit bacteria by adsorbing to oral tissues and disrupting bacterial cell membranes [14]. CPC also prevents bacterial co-aggregation and biofilm formation, offering potential in dental restorative materials [15]. 

Efforts to enhance the antibacterial properties in RBDCs mainly focus on releasing low-molecular-weight antibacterial agents such as zinc and silver ions [16], iodine, and chlorhexidine [16]. Chlorhexidine (CHX), a widely used leachable antibacterial agent, leaves voids affecting mechanical properties [17]. An addition of 0–5% zinc oxide to RBDC reduced bacterial growth, maintained physical strength, but significantly decreased depth of cure due to increased opacity [18]. Antibacterial monomers, namely Methacryloyloxydodecylpyridinium bromide, in RBDCs maintain activity for 90 days in water [19]; however, they exhibit primarily bacteriostatic and less potent antimicrobial effects compared to RBDCs containing soluble antibacterial compounds. Another drawback includes their reduced antibacterial activity due to surface protein adsorption [20]. The incorporation of silver nanoparticles (0.5–10%) in RBDCs reduces bacterial growth, but may cause discoloration [16]. RBDCs containing quaternary ammonium polyethyleneimine exhibit antibacterial function for at least one month, though safety concerns about polyethyleneimine persist [21].

Urethane dimethacrylate quaternary ammonium compound containing composite resin exhibits significant antibacterial properties against *Streptococcus mutans* while showcasing biocompatibility, an adequate flexural strength, and a modulus suitable for potential stress-bearing and caries-inhibiting restorations [22]. 

Resin composites with 18% quaternary ammonium dimethacylate (QADM) exhibited approximately half the biofilm CFU, metabolic activity, and acid production compared to those without QADM [23]. Notably, the sustained antibacterial efficacy of novel nanocomposites containing QADM was evident, even following water aging for 30, 90, and 180 days [24]. Incorporating dimethylaminohexadecyl methacrylate into self-cured resin proved to be an effective means of inhibiting biofilm formation by *Streptococcus mutans* and *Candida albicans*. Nevertheless, this novel polymer had detrimental effects on cytotoxicity, as well as the physical and mechanical properties assessed in the study [25]. 

All the bactericidal agents discussed above have a drawback or restriction that essentially prevents their use while developing antibacterial dental RBDCs. CPC has been used as a powerful antimicrobial agent and has been frequently employed in various over-the-counter products, including buccal tablets [26], sanitizer [27], disinfecting liquids [28], cleaning agents for eatables [29], nasal sprays [30], mouthwashes [31], and toothpastes [32]. Even though there are not much data present in the literature regarding the incorporation of CPC into RBDCs, many other studies have confirmed its antimicrobial effect in different dental materials, for instance varnishes [33], adhesives [34,35], orthodontic primers [36], root canal sealers [37], acrylic resin [38], and cements [39]. In 2019, Kenya Matsuo et al. [34] demonstrated, with the help of optical density and SEM, that CPC montmorillonite adhesives significantly inhibited the growth of *Streptococcus mutans* biofilms. Tanvi Verma et al. [38], in their study, analyzed the length of CPC release from modified acrylic and its antibacterial function. The authors observed that an increase in CPC concentration in the acrylic increased its antimicrobial activity. Similarly, an innovative poly(methyl methacrylate) cement with antibacterial agent CPC—montmorillonite in two filler sizes at various doses—was investigated by Yuya Yamamoto et al. [40], in which they were able to achieve persistent anti-biofilm activity in the poly(methyl methacrylate) cement by incorporating 5–7.5 %wt of CPC, while simultaneously preserving mechanical strength and bonding performance. While CPC has found its way into a few dental materials, a notable void persists in the literature regarding its incorporation specifically into RBDCs. This gap in research underscores the unique and unexplored dimensions that our study seeks to explore in the context of RBDCs. Therefore, in this study, we aimed to investigate the effect of CPC addition on the antibacterial and surface hardness characteristics of two commercial RBDCs.

## 2. Materials and Methods

### 2.1. Experimental RBDCs Preparation

**Figure 2:**
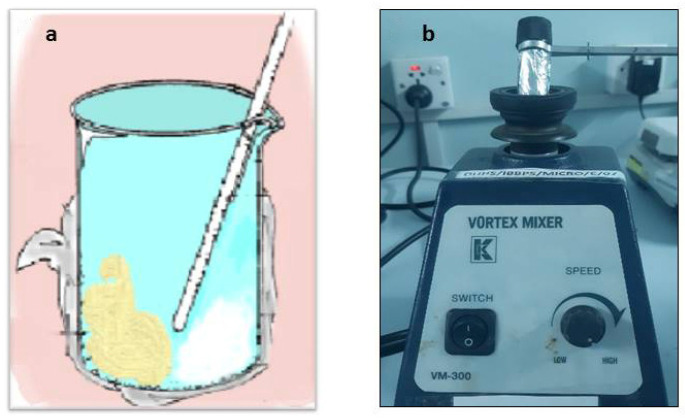
(**a**) Image depicts the manual mixing of RBDC (brown) and CPC (white) in a glass beaker covered with aluminum foil, using a glass rod. (**b**) Mixing of RBDC and CPC within a Scintillation Vial, Shielded by Aluminum Foil, on the Vortex Machine.

A total of four experimental groups were prepared by blending 2% and 4% by wt of CPC (Sigma Aldrich, St. Louis, MO, USA) into two RBDCs, microhybrid Filtek Z250 universal restorative (3M ESPE, St. Paul, MN, USA) and Filtek Z350 XT flowable restorative (3M ESPE, St. Paul, MN, USA). The compositions of both RBDCs are given in Table 1. The RBDC and CPC powders were weighed using analytical balance (Mettler Toledo AL204, Mississauga, ON, Canada, accuracy 0.1 mg) and mixed in a 50 mL beaker by manual spatulation in a dark room under a controlled environment (23 ± 1 °C and 55% humidity) by stirring with a glass rod vigorously for about 1 min to produce a cohesive RBDC paste (Figure 2a). Moreover, the laboratory vortex mixer (Gemmy vortex mixer; VM-300, Taipei, Taiwan) was also used to thoroughly mix the RBDCs for another 1 min at a speed of 3150 rpm in a 5 mL scintillation vial (Figure 2b). The glass vials and beaker were both coated with aluminum foil to offer shielding from ambient light. In addition, two groups of specimens from the same commercial RBDCs without the addition of CPC were used as comparative controls.

### 2.2. Specimen Fabrication

**Figure 3:**
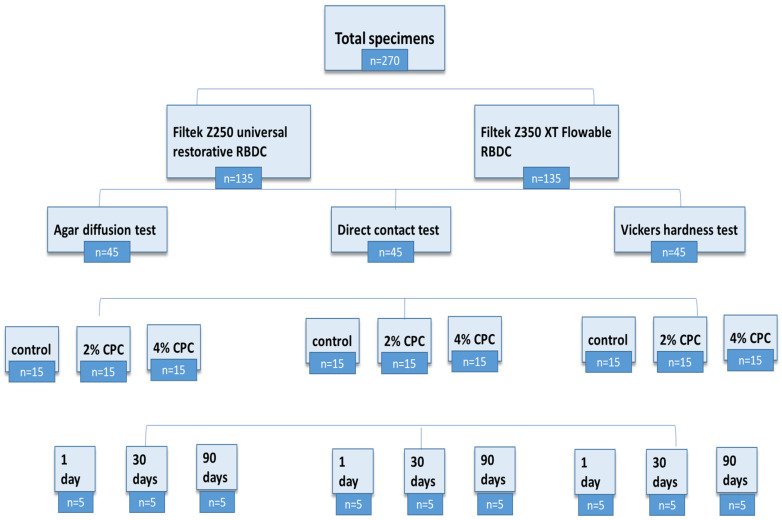
Flow chart showing sample size distribution.

A total of two hundred and seventy (*n* = 270) (Figure 3) specimens were prepared using circular brass molds (2 mm thickness × 6 mm diameter) [41]. The mold was set on top of a 50 µm thick mylar strip that was laid out on a glass slab in accordance with the ISO standard 4049 [42]. Using a sterile stainless steel spatula, the RBDC material was slightly overfilled into the mold. Another piece of mylar strip was used to cover the mold to avoid the entrapment of an oxygen layer in the material bulk [43]. The excess material was squeezed out completely by applying pressure with another glass slab. Photopolymerization was carried out using LED curing light (Starlight pro Mectron, Italy, intensity 1400 mW/cm^2^, optical wavelength 440–480 nm) from the top surface for 40 s [39]. The tip of the light curing device had a diameter of 10 mm. The specimens were polished manually with abrasive paper sequentially to increase grain size (300–1200 grit SiC). All test specimens were stored in sterile Eppendorf tubes filled with distilled water and were placed for 1 day (*n* = 90), 30 days (*n* = 90), and 90 days (*n* = 90) in an incubator (Esco IFA-54-8, Singapore) at 37 °C. Moreover, the distilled water of each specimen group was replaced every 24 h in order to prevent the accumulation of leached RBDC content in the Eppendorf tubes [44].

### 2.3. Scanning Electron Microscopy (SEM)

For SEM, the specimens were coated with a thin layer of platinum using a sputter coater (Jeol—JEC-3000FC/Auto Fine Coater, JEOL, Peabody, MA, USA). The SEM analysis was performed using a Scanning electron microscope (JSM-IT100, JEOL, Peabody, MA, USA) with an accelerating voltage from 500 kV to 30 kV at 4000× and 10,000× magnifications. 

### 2.4. Antibacterial Testing

A pure culture of *Streptococcus mutans* ATCC^®^ 25175^™^ was used while gram staining was performed for microscopic confirmation of the purity of the bacteria. The tryptic soy broth (TSB) medium (Sigma, St. Louis, MO, USA) containing bacterial inoculum was incubated at 37 °C for 24 h in a 5% CO_2_ incubator (ESCO CCL-050B-8, Singapore). The bacterial suspensions were adjusted to a turbidity of 0.5 McFarland standard, equivalent to 1.5 × 10^8^ CFU/mL, using a Multiskan Sky Microplate Spectrophotometer (Thermo Scientific™, Waltham, MA, USA). 

### 2.5. Agar Diffusion Test

A total of ninety (*n* = 90) composite specimens were tested for their antibacterial activity with an agar diffusion test. A standardized bacterial suspension (200 µL) was uniformly spread onto petri plates containing Muller–Hinton agar (Sigma, St. Louis, MO, USA). Using a sterile cotton swab, the agar plates were streaked by spreading inoculum over the entire agar surface evenly while the plate was held at approximately 60°. With the help of sterile metal tweezers, the specimen discs were placed by firmly pressing down onto the inoculated agar plate’s surface with a 2 cm distance from each other. Four discs were placed on each plate, including two experimental RBDC discs, a positive control, and a negative control disc. For the positive control, 0.2% chlorhexidine gluconate mouthwash (Protect, Karachi, Pakistan) was poured on filter paper discs with dimensions matching the RBDC discs. The concentration applied was 0.075 ppm. The plates were then placed in a CO_2_ incubator for 48 h set at 37 °C and 5% CO_2_. After incubation, the diameter of the inhibition zone around each specimen was measured using vernier calipers in two perpendicular locations [2].

### 2.6. Direct Contact Inhibition Test

Ninety composite specimens (*n* = 90) underwent testing for antibacterial activity using the direct contact inhibition test. Each specimen (*n* = 5) from the experimental and control RBDC groups was placed in the Eppendorf tube, inoculated with 10 µL of the bacterial suspension, and incubated for an hour at 37 °C to allow the liquid to evaporate and bring the bacteria into direct contact with the test specimens. The tubes were then filled with 300 µL of freshly made, autoclaved tryptic soy broth (TSB) and left to incubate for 48 h in a 5% CO_2_ incubator (CO_2_ incubator (ESCO CCL-050B-8, Singapore). The bacterial sample was then serially diluted using the miles and misra method [9]. The bacterial suspension from the original specimen-containing Eppendorf tube was transferred to subsequent 1.5 mL Eppendorf tubes containing plain PBS, resulting in a series of dilutions. Specifically, 100 µL of the bacterial suspension was added to each of the seven Eppendorf tubes, which contained 900 µL of plain PBS. Four droplets per sample dilution were pipetted onto the marked sections of agar in each petri dish. The petri dishes were then incubated in a 5% CO_2_ incubator (ESCO CCL-050B-8, Singapore) at 37 °C for 24 h. The sample dilution that produced around thirty colonies per drop was chosen. At least four drops were used to obtain an average count. The formula below was used to compute the bacterial CFU.

$$CFU=\frac{col\times dilution}{volume}$$

Here, ‘*CFU*’ represents the colony-forming units, ‘*col*’ represents the number of colonies counted, ‘*dilution*’ for the dilution factor used, and ‘*volume*’ represents the volume plated on the agar plate.

### 2.7. Vickers Microhardness Test

Microhardness testing using a Vickers tester involved a total of ninety RBDC specimens (*n* = 90). Three readings at random positions for each specimen, approximately at the center, with the use of the Vickers tester (Indentec ZHV, Zwick/Roell Indentec, Worcestershire, UK) with a 100 g load and 15 s loading time, were taken [45]. The Vickers hardness test or diamond pyramid test is a microindentation technique. The indenter creates a square indentation on the surface of the specimen. The unit of hardness given by the test is known as the Vickers hardness number (VHN).

### 2.8. Data Analysis

Data were entered and statistical analysis was performed using the SPSS v21 software (IBM SPSS). Mean and standard deviations were reported for outcome variables. Three-way ANOVA tests were also applied for the determination of interactions among different times, concentrations, and type of RBDC used for the agar diffusion test, direct contact test, and Vickers hardness test. Pair-wise inter-group and section comparisons by post hoc LSD with Bonferroni correction with *p*-value were conducted. To control type I errors intended for multiple comparisons, Bonferroni correction was used. The overall model level of significance was set at *p* < 0.05.

## 3. Results

### 3.1. Scanning Electron Microscopy

**Figure 4:**
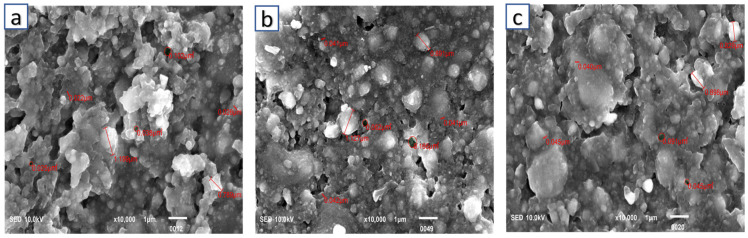
Scanning electron microscopic image of Z250 universal, (**a**) control, (**b**) 2 %wt-CPC experimental RBDC, and (**c**) 4 %wt-CPC experimental RBDC.

The surface morphology/structure of the Z250 universal RBDC of the control, 2%wt, and 4%wt is shown in Figure 4. The surfaces of all specimens were smooth and free of cracks. Massive agglomerates of particles of different sizes were seen in all images. The particle sizes of glass fillers varied from a much smaller 0.022 µm to a much larger 1.199 µm (Figure 4a). The addition of CPC into the universal RBDC led to a somewhat similar morphology and structure as compared to the control. A uniform distribution of particles could be seen. The structure of CPC was not clearly visible in both experimental universal RBDCs. However, a few pores were seen in the structure of the experimental RBDC with sizes ranging from (0.045–0.091 µm) (Figure 4b,c). 

**Figure 5:**
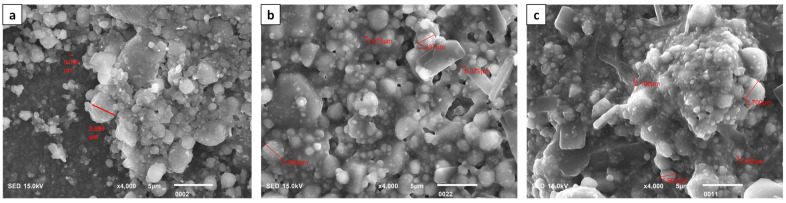
Scanning electron microscopic image of Z350 XT flowable, (**a**) control RBDC, (**b**) 2%-CPC experimental RBDC, and (**c**) 4%-CPC experimental RBDC.

In Figure 5, SEM micrographs show the surface morphology/structure of the Z350 XT flowable RBDC without the incorporation of CPC, the 2 %wt-CPC experimental RBDC, and the 4 %wt-CPC experimental RBDC, respectively. The particle sizes of glass fillers ranged from the smallest at 0.030 µm to the largest at 2.693 µm (Figure 5), and the particles of CPC were clearly seen, appearing as a crystal shape of a parallelogram overlapping and clustering with filler particles (Figure 5b,c). A uniform distribution of CPC particles into the RBDC structure was observed, however, the typical structure of the flowable RBDC was preserved. 

### 3.2. Agar Diffusion Test

**Figure 6:**
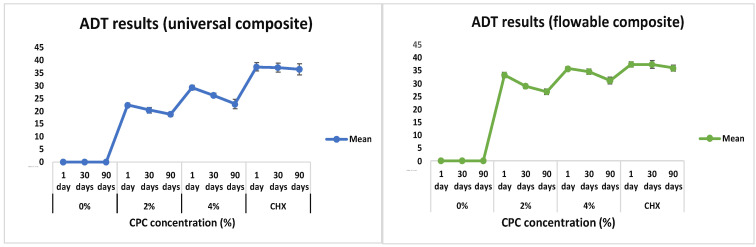
Graphical representation of agar diffusion test results for universal and flowable RBDCs.

**Figure 7:**
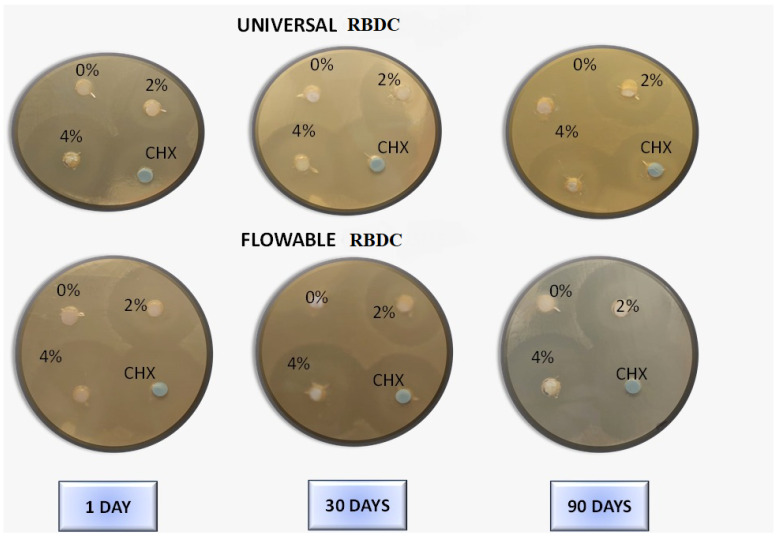
Agar disc diffusion assay for evaluation of antibacterial activity of varied concentrations (0 %wt, 2 %wt, and 4 %wt) of CPC incorporated in universal and flowable RBDCs following different storage times against *Streptococcus mutans*.

Graphical and pictorial representations of the antibacterial activity of the RBDCs with and without modifications of CPC at 2 %wt and 4 %wt concentrations are shown in Figure 6 and Figure 7. Without the incorporation of CPC (0%wt concentration), the RBDC demonstrated no discernible antibacterial activity. Notably, both the universal and flowable RBDCs exhibited the highest efficacy with 4 %wt CPC across all time intervals (1, 30, and 90 days) (Table 2). Statistical analysis underscored a significant difference between the control group (0 %wt CPC) and the RBDCs containing 2 %wt and 4 %wt CPC (*p*-value = 0.001). The data further demonstrated a positive correlation between an increasing CPC concentration and enhanced antibacterial activity. However, it is noteworthy that the antibacterial activity showed a decline over time. In the context of the agar diffusion test for the flowable RBDC, no significant difference was observed between 4 %wt CPC and the positive control (CHX) (*p*-value = 0.577) (Table 2). 

### 3.3. Direct Contact Inhibition Test

**Figure 8:**
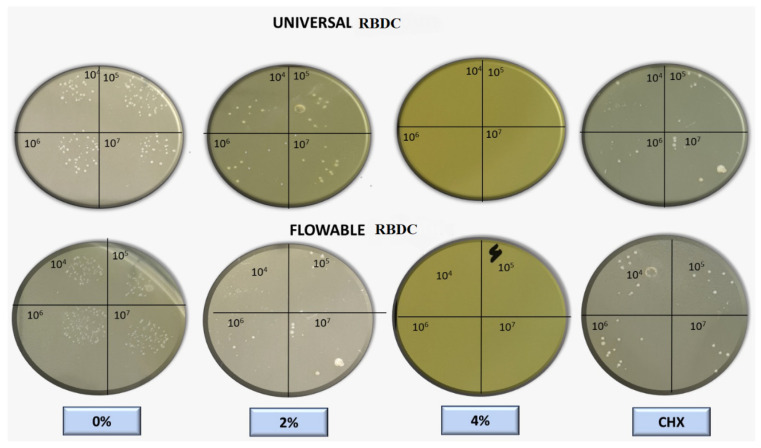
Macroscopic observation of colony-forming units for universal and flowable RBDCs on direct contact test against *Streptococcus mutans*.

**Figure 9:**
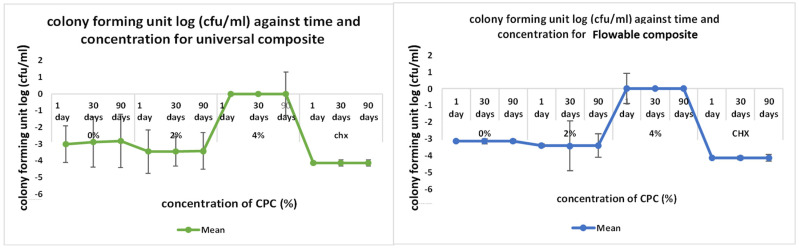
Graphical representation of direct contact test results for universal and flowable RBDCs.

The outcomes of the direct contact inhibition test assessing the bacterial activity of the experimental RBDCs containing 2 %wt and 4 %wt CPC revealed a noteworthy reduction in colony-forming units (CFU) compared to the control group without CPC (0 %wt CPC) incorporation (*p*-value < 0.001) (Figure 8 and Figure 9). This reduction in CFU count was consistently observed across both the universal and flowable RBDCs at all tested storage durations (1 day, 30 days, and 90 days), establishing a statistically significant difference between the control and the 2 %wt-CPC and 4 %wt-CPC groups (*p*-value = 0.000) (Figure 8 and Figure 9). Interestingly, the comparison between the 4 %wt-CPC and CHX groups demonstrated an insignificant difference in CFU count (*p*-value > 0.05) (Table 3). Importantly, over time, there was no significant change in the CFU count for both the 4 %wt-CPC and CHX groups (*p*-value > 0.05). However, with time, no difference in the colony-forming unit count (CFU) was found (*p*-value > 0.05) (Table 3).

### 3.4. Surface Hardness

The Vickers Microhardness values of the two RBDCs, both control and modified with CPC- 2 %wt and 4 %wt, are presented in Table 4. The addition of up to 2 %wt CPC to the RBDCs did not result in a significant alteration in their hardness values (*p*-value > 0.01). However, a notable decline in mean hardness was observed for the 4 %wt CPC group, showing statistical significance from 1 day to 30 days and 90 days of storage (*p*-value = 0.001). The hardness measurements of the control and experimental universal RBDCs ranged between 54.8 and 72.1 VHN, while the flowable RBDCs exhibited hardness values within the range from 32.9 to 48.1 VHN. 

## 4. Discussion

In this study, the antibacterial efficacy of CPC-modified RBDCs was evaluated for a period of 90 days to predict the long-term antibacterial effect of these RBDCs against *Streptococcus mutans*. According to the findings of the agar diffusion test (ADT) and direct contact inhibition test (DCT), it was found that the antibacterial properties of both types of dental RBDCs were noticeably improved within the first 24 h of incorporating CPC at concentrations of 2 %wt and 4 %wt, with a slow and gradual loss of antibacterial properties over intervals of 30 days and 90 days, as indicated by the zone of inhibition surrounding these discs in the ADT and the CFU count in the DCT. However, antibacterial activity was sustained throughout the aging period. On the other hand, dental RBDCs without any incorporation of CPC showed no antibacterial activity at any time period. These results are consistent with other studies that have investigated the antibacterial activity of CPC in other dental materials. 

An SEM assessment of the RBDCs was performed to observe the morphology and distribution of CPC particles in the experimental RBDCs compared with the control. Between the experimental and control RBDCs, no significant changes in the microstructure after modification with CPC were observed, except a few porosities. This may be attributed to the interaction of CPC with the filler particles in the RBDC, which can affect the chemical interactions between the polymer matrix and the filler particles, inhibiting the polymerization reaction, which can result in the formation of pores in the RBDC. It is important to note that these are just possibilities, and further research is required to confirm the exact reason why CPC causes pores in RBDCs. 

In acrylic resins modified with CPC, Tanvi Verma et al. [38] observed that antibacterial activity was found in all CPC-containing acrylic resin specimens, but its effectiveness decreased with time. Al-Musallam et al. [35] confirmed the maintenance of antimicrobial activity in CPC-modified orthodontic adhesive resin against *Streptococcus mutans* discs after being stored in distilled water for a 196-day testing period, with a small decline in the zones of bacterial inhibition as the aging time increased, which is in agreement with our findings. The observed decrease in antibacterial activity over time may be attributed to the leaching of CPC from the experimental RBDCs during the water aging of the specimens. They concluded that, as the concentration of CPC increased from 2.5 %wt to 5 %wt and 10 %wt, the amount of CPC release also increased, similar to our results. In a study conducted by Naoka Namba et al. [46], *Streptococcus mutans* growth was strongly reduced on the surface of a 3 %wt-CPC-containing resin coating as compared to a non-CPC resin coating. This is in agreement with our results, in which the 4 %wt-CPC RBDCs showed the highest bacterial inhibition when coming into contact with *Streptococcus mutans* in the direct contact inhibition test, sustaining their antibacterial activity until 90 days. It is essential to note that the findings are probably consistent with our study because the CPC used in our research and this study had comparable chemical characteristics, purity, and composition. The fact that both studies used different CPC concentrations but still discovered that increasing the concentration led to higher antibacterial activity indicates that the antibacterial effect of CPC is concentration-dependent, regardless of the specific concentration used; this might be because the antibacterial mechanism of CPC is well-established and consistent across different products.

Direct restorative composite materials utilized in dentistry generally have a Vickers hardness range from 40 VHN to over 100 VHN [47,48]. All the measured values in our study were found to be within the typical range, with the exception of the 4 %wt CPC flowable RBDC after 30 days and 90 days of aging. The degree of polymerization can be inferred from the hardness of the RBDCs [49].

Our SEM analysis identified the presence of scattered voids in the RBDCs containing 4 %wt CPC. This observation is likely attributable to the manual mixing process. This aligns with the findings reported by Kumar and Shortall. [50], who observed increased porosity in hand-spatulated resin-based composites (RBC), a phenomenon expected due to the incorporation of air during the mixing process. 

Consequently, the outcomes of our investigation indicate that the incorporation of 2 %wt CPC into flowable and universal RBDC yielded optimal hardness levels for a period of 90 days. It is important to consider that these studies are laboratory-based and conducted under controlled conditions, so more research is needed to evaluate the long-term clinical performance and effectiveness of CPC-incorporated RBDCs in real-life scenarios.

Tsubasa N. et al. [51] added 2 %wt CPC into tissue conditioner and found no significant difference between it and its control counterpart, which supports the results of our study. The fact that both studies employed a similar concentration of CPC (up to 2 %wt) suggests that this level may not be substantial enough to induce a significant alteration in the microhardness of the materials under investigation. Hence, this may explain why the results of the two studies were similar. An RBDC with from 0.5 to 1.0 wt% of calcium fluoride resulted in an increase in microhardness when stored in dry and wet conditions, as conducted by Mitwalli H. et al. [52]. The contrasting results of this study compared to our study might be due to the type and concentration of antimicrobial agent used. Since antimicrobial agents vary in their chemical compositions and properties, they may also have different effects on the properties of RBDCs. The strength of the impact of the antimicrobial agent on the physical properties of the RBDC can also be influenced by its concentration.

This study has some limitations that need to be considered when analyzing the results, such as the use of only one type of bacteria to investigate the antimicrobial activity of the RBDCs. This may not provide a complete understanding of the antibacterial efficacy of the RBDCs against a range of oral pathogens. It is an in vitro study, which may not accurately reflect the clinical real-life conditions in the oral cavity. This study’s strengths lie in the use of commercially available dental RBDCs, offering real-world applicability for dental practitioners. Examining two CPC concentrations enhances our understanding, while a 90-day evaluation ensures insight into durability and stability, including potential adverse effects. Analyzing two commercial RBDCs with varied viscosities provides a comprehensive view, and the study’s novelty adds significant value, as no prior research has explored the same question or methodology.

Further exploration of these CPC-incorporated RBDCs is warranted to assess various mechanical and physical variables, including flexural strength, diametral tensile strength, water sorption and water solubility, and creep, etc. This extended research will contribute to a more comprehensive understanding of the composite material, paving the way for its potential applications in dental practice and commercial settings. Additional studies can be carried out to evaluate the impact of CPC on dental RBDCs in vivo. Clinical trials can provide a more comprehensive understanding of the impact of CPC on dental RBDCs, including the impact on oral health, patient comfort, and durability over time. 

## 5. Conclusions

Within the limitations of this study, it can be concluded that the RBDCs modified with CPC demonstrated a significant decrease in bacterial colonies in the direct contact inhibition test and a larger zone of inhibition in the agar diffusion test against the *Streptococcus mutans* strain, indicating its high initial antibacterial effectiveness. The successful combination of the antibacterial effect with micromechanical properties was achieved for a CPC concentration of 2 %wt. These materials exhibited hardness values like the base material, as well as significant antibacterial activity until the end of the 90-day storage period. The comparable hardness values observed between the control and the RBDCs modified with 2% CPC suggest that the polymerization process of the RBDCs remained unaffected by the addition of CPC up to the 2% concentration level.

## Figures and Tables

**Table 1:** Composition of commercial RBDCs used in this study.

RBDC Type	Manufacturer	Batch No.	Classification	Resin	Filler	Particle Size
Filtek Z250^™^	3M ESPE; USA	NA34552	Microhybrid	BisGM A UDMA BisEMA TEGDM A	Zirconia/silica; 84.5 %wt, 60 volume%	0.01 µm to 3.5 µm
Filtek Z350 Flow^™^	3M ESPE; USA	NA73575	Nanohybrid	BisGMA BisEMA TEGDM A	Zirconia/silica and silica; 65 %wt, 55 %vol	0.6 to 20 µm

Bis-GMA: bisphenol A diglycidyl ether dimethacrylate; UDMA: urethane dimethacrylate; Bis-EMA: bisphenol A polyethylene glycol diether dimethacrylate; and TEGDMA: triethylene glycol dimethacrylate.

**Table 2:** Zone of inhibition data (in mm) for universal and flowable RBDCs on comparison with different storage times at different concentrations of CPC.

Universal RBDC				Flowable RBDC			
Time (Day)	1	30	90	Time (Day)	1	30	90
Conc.				Conc.			
0%	00 ^a^	00 ^a^	00 ^a^	0%	00 ^a^	00 ^a^	00 ^a^
2%	22.4± 0.8 ^b^	20.4± 1.1 ^b^	18.8± 0.8 ^b^	2%	33.4± 1.1 ^b^	29.0 ± 2.0 ^b^	26.8 ± 1.3 ^b^
4%	29.2± 0.8 ^c^	26.2± 0.8 ^c^	22.8± 1.9 ^c^	4%	35.8 ± 0.8 ^c^	34.6 ± 1.1 ^c^	31.2 ± 1.3 ^c^
CHX	37.4 ± 1.7 ^c^	37.1 ± 1.8 ^d^	36.5 ± 2.2 ^d^	CHX	37.4 ± 1.1 ^c^	37.5 ± 1.5 ^d^	37.5 ± 1.5 ^d^

Superscripts with dissimilar alphabets down columns show statistically significant difference (*p* < 0.05).

**Table 3:** Mean colony-forming unit count for universal and flowable RBDCs for comparison with different storage times at different concentrations of CPC.

Universal RBDC				Flowable RBDC			
Time (Day)	1	30	90	Time (Day)	1	30	90
Conc.				Conc.			
0%	7.5 × 10^−4^ ± 0.15 ^a^	7.3 × 10^−4^ ± 0.1 ^a^	7.4 × 10^−4^ ± 0.1 ^a^	0%	1.0 × 10^−3^ ± 1.1 ^ac^	1.5 × 10^−3^ ± 1.1 ^a^	1.3 × 10^−3^ ± 1.1 ^a^
2%	4.0 × 10^−4^ ± 1.5 ^b^	3.9 × 10^−4^ ± 0.7 ^b^	4.0 × 10^−4^ ± 0.9 ^b^	2%	3.5 × 10^−4^ ± 1.3 ^b^	3.6 × 10^−4^ ± 1.3 ^a^	3.7 × 10^−4^ ± 1.3 ^a^
4%	00 ^c^	00 ^c^	00 ^c^	4%	00 ^a,c^	00 ^c^	00 ^c^
CHX	7.3 × 10^−5^ ± 0.1 ^c^	7.3 × 10^−5^ ± 0.2 ^c^	7.3 × 10^−5^ ± 0.2 ^c^	CHX	7.3 × 10^−5^ ± 0.1 ^a^	7.0 × 10^−5^ ± 0.1 ^a^	7.2 × 10^−5^ ± 0.1 ^a^

Superscripts with dissimilar alphabets down columns show statistically significant difference (*p* < 0.05).

**Table 4:** Mean Vickers hardness number for universal and flowable RBDCs for comparison with concentrations of CPC at different times.

Universal RBDC				Flowable RBDC			
Time (Day)	1	30	90	Time (Day)	1	30	90
Conc.				Conc.			
0%	72.1 ± 1.2 ^a^	71.8 ± 1.1 ^a^	63.4 ± 1.5 ^a^	0%	48.1 ± 0.7 ^a^	46.3 ± 0.8 ^a^	41.2 ± 0.8 ^a^
2%	71.2 ± 1.1 ^a^	70.9 ± 1.5 ^a^	59.0 ± 0.7 ^b^	2%	46.2 ± 0.8 ^a^	45.7 ± 1.1 ^a^	35.9 ± 0.9 ^b^
4%	70.1 ± 1.2 ^a^	60.0 ± 0.8 ^b^	54.8 ± 1.1 ^c^	4%	43.0 ± 2.0 ^b^	41.2 ± 0.8 ^b^	32.9 ± 0.9 ^c^
CHX	72.1 ± 1.2 ^a^	71.8 ± 1.1 ^a^	63.4 ± 1.5 ^a^	CHX	48.1 ± 0.7 ^a^	46.3 ± 0.8 ^a^	41.2 ± 0.8 ^a^

Superscripts with dissimilar letters down columns indicate statistically significant difference (*p* < 0.05).

## Data Availability

The data presented in this study are available on the request from the corresponding author.
